# Thoracic Spinal Anesthesia With Erector Spinae Plane Block Versus Conscious Sedation for Medical Thoracoscopy: A Comparative Pilot Study

**DOI:** 10.7759/cureus.101416

**Published:** 2026-01-13

**Authors:** Archana Baburao, Parinita Suresh, Sudeeksha P, Karthik GS, Thirthashree K, Aleena M Mathew, Mohammed Munavvar

**Affiliations:** 1 Respiratory Medicine, Rajarajeswari Medical College and Hospital, Dr. M.G.R. Educational and Research Institute, Bangalore, IND; 2 Pulmonary Medicine, Rajarajeswari Medical College and Hospital, Bangalore, IND; 3 Anesthesiology and Critical Care, Rajarajeswari Medical College and Hospital, Bangalore, IND; 4 Respiratory Medicine and Interventional Pulmonology, Lancashire Teaching Hospitals, University of Central Lancashire, Preston, GBR

**Keywords:** conscious sedation, erector spinae plane block, medical thoracoscopy, quality of recovery, thoracic spinal anesthesia

## Abstract

Background and objective

Medical thoracoscopy (MT) is associated with significant postoperative pain of varying intensity and duration, leading to significant morbidity. Our study compared the efficacy of thoracic spinal anesthesia (TSA) combined with erector spinae plane block (ESPB) with conscious sedation for MT using a patient-centered outcome measure.

Methods

This is a non-randomized prospective comparative pilot study wherein 36 patients undergoing MT were assigned alternately to receive TSA with ESPB or conscious sedation. Conscious sedation was administered with fentanyl and midazolam in graded doses with local instillation of lignocaine 2%. TSA was administered at the T6-T7 level with 25 mcg of fentanyl and 0.5% levobupivacaine (1.5 ml), followed by ESPB with 0.25% levobupivacaine (10 ml). The primary outcome was to compare the efficacy of ESPB with conscious sedation in terms of quality of recovery. The secondary outcome was to compare the time to administration of the first analgesic post procedure, total postoperative opioid consumption (mg) at 24 hours, and duration of the procedure. Intraoperative hemodynamic stability and block-related and post-procedure complications were also assessed.

Results

The total Quality of Recovery-15 (QoR-15) score was 112.83 ± 27.16 vs. 65.78 ± 22.13 (p = 0.0001), time to first analgesic was 11.69 ± 6.93 hours vs. 2.39 ± 1.69 hours (p = 0.0001), opioid consumption was 50 ± 10 vs. 75 ± 15 (p = 0.001), and the mean duration of the procedure was 64.72 vs. 93.89 minutes (p = 0.0001) in the ESPB group and the conscious sedation group, respectively. No block/anesthesia-related complications were reported in either group.

Conclusion

The combination of TSA with ESPB can be a novel, effective, and safe anesthetic technique for MT, offering better quality of recovery, improved postoperative analgesia, shorter procedure duration, and reduced perioperative morbidity.

## Introduction

Medical thoracoscopy (MT) is considered the gold standard for undiagnosed pleural effusions. It can be performed in an endoscopy suite under local anesthesia/conscious sedation using rigid/semi-rigid instruments, which makes it considerably less invasive and less expensive than video-assisted thoracoscopic surgery (VATS), which is performed in an operating room under general anesthesia with selective intubation [1]. The diagnostic yield with MT biopsy is close to 98%, with a sensitivity of 93% for malignant and 98% for tuberculous pleural effusions in endemic countries [2]. MT is a relatively safe procedure with low mortality rates of 0.01-0.24% and a major complication rate of 1.8% [3]. Local anesthesia with moderate sedation, widely termed “conscious sedation,” is the anesthetic technique of choice for MT, but it does not provide anesthesia to the parietal pleura where biopsies are obtained, leading to significant pain both during and post procedure, patient discomfort, and difficult operating conditions [4]. Post-thoracoscopic pain has emerged as the most common complication affecting 33% of patients undergoing MT [5].

Various strategies, such as combining benzodiazepines with opioids, titrated propofol infusions, and intrapleural lignocaine instillation, have been employed to improve pain management. More recently, regional anesthetic techniques, including erector spinae plane block (ESPB), paravertebral block, and intercostal nerve blocks, are being investigated. ESPB is an interfascial plane block utilized as regional anesthesia in thoracic surgery, first described by Forero et al. in 2016 [6]. It has been shown to decrease sedation requirements during the procedure and significantly alleviate pain severity both during and post procedure. ESPB is safe, with no reported adverse effects. Furthermore, the ease of identifying the erector spinae plane (ESP) using ultrasonography (USG) makes it an accessible and feasible method for enhancing MT. In our study, thoracic spinal anesthesia (TSA) was preceded by ESPB to extend the duration of analgesia and reduce morbidity during the postoperative period. Studies have compared regional anesthesia techniques in patients undergoing VATS and other thoracic surgeries [7,8]. Our study is one of the first to compare conscious sedation (local anesthesia with moderate sedation) and TSA with ESPB in MT.

## Materials and methods

This non-randomized prospective comparative pilot study was conducted at the Department of Respiratory Medicine, Rajarajeswari Medical College and Hospital, Dr. M.G.R. Educational and Research Institute, Bangalore, India, from April 2024 to October 2024. Patients with unilateral undiagnosed exudative pleural effusion were enrolled in the study and were screened for inclusion and exclusion criteria. Informed written consent was taken from all subjects, and ethical clearance was obtained from the Institutional Ethics Committee (RRMCH-IEC/229/2024).

Patients who were 18 years of age or older, with American Society of Anesthesiologists (ASA) grade 1-3, unilateral undiagnosed exudative pleural effusion undergoing MT, and willing to answer the Quality of Recovery-15 (QoR-15) questionnaire [9] were included in the study. The QoR-15 questionnaire is open access for non-commercial use as long as an appropriate citation is given (Copyright by the American Society of Anesthesiologists). Permission for its use in this study was formally obtained from Dr. Paul Myes, the original developer of the questionnaire. Patients with pre-existing infection at the block site, contraindication to regional anesthesia, allergy to lidocaine, levobupivacaine, or other local anesthetics, pre-existing chronic pain or cognitive dysfunction that would impede accurate assessment of post-procedure QoR, and analgesia were excluded from the study. Based on the inclusion and exclusion criteria, 36 patients were assigned alternatively to one of the two groups, i.e., the TSA-ESPB group and the conscious sedation group.

The conscious sedation group included 18 patients who received conscious sedation with Inj. fentanyl 1-2 mcg/kg and Inj. midazolam 1-2 mg slow IV in graded doses throughout the procedure with local instillation of lignocaine 2% (20 mg/ml) at a dose of up to 3 mg/kg. The patients were spontaneously breathing with oxygen via a facemask.

The TSA-ESPB group included 18 patients who were given TSA with ESPB by an anesthesiologist as follows. Under aseptic precautions, TSA was performed first with the patient in the sitting position, with a 25-G Quincke needle at the level of the T6-T7 intervertebral space. Once there was free-flowing, clear CSF, 25 mcg of Inj. fentanyl and 1.5 ml of Inj. 0.5% isobaric levobupivacaine was administered intrathecally. Ten minutes after the placement of the initial spinal anesthetic, once an adequate level of the block was achieved, patients were positioned in the lateral position. Counting down from the C7 vertebra, the T6 vertebra was identified. Using the in-plane technique, the linear probe placed in a sterile cover (SonoSite M-Turbo, FUJIFILM SonoSite, Inc., Bothell, WA) was moved until the erector spinae muscle and transverse process were visualized on the affected side of the hemithorax. A Tuohy needle was directed toward the transverse process underneath the fascia of the erector spinae muscle on the side of the procedure. Correct needle-tip position was confirmed by the presence of a linear spread of 2-3 ml of normal saline between the transverse process and the erector spinae muscle group, following which Inj. levobupivacaine 0.25% 10 ml was injected under ultrasound guidance. The patient was spontaneously breathing with oxygen via a facemask and did not receive any additional sedation or analgesics.

In both groups, MT was done using a rigid thoracoscope (10 mm diameter scope with 6 mm working channel; Karl Storz KG, Tuttlingen, Germany). All the patients underwent parietal pleural biopsy. Breaking the loculations and adhesiolysis were done as required. The patient's vitals were continuously monitored during the procedure. Episodes of hypotension were treated with Inj. phenylephrine 25 mcg boluses. In case of an emergency, for quick conversion into general anesthesia, a laryngoscope, an endotracheal tube, anesthetic agents, and other resuscitation equipment were kept ready. Post procedure, patients were transferred to the post-anesthesia care unit (PACU) and then to the ward when deemed fit, as suggested by the Aldrete score [10]. Aldrete scoring system is free to use and reproduce for clinical and research purposes without permission, as long as an appropriate citation is given. Patients were prescribed Inj. tramadol 50 mg IV as required for post-procedure pain in PACU and oral Tramadol as required in the ward unless contraindicated. Ondansetron 8 mg IV/4 mg oral was prescribed as required for nausea and vomiting post procedure.

The primary outcome was to compare the efficacy of TSA-ESPB vs. conscious sedation in terms of quality of recovery post procedure. Post procedure, all patients were subjected to the QoR-15 questionnaire at 24 hours to assess 15 parameters across five domains (pain, physical comfort, physical independence, psychological support, and emotional status), each being graded from 0 to 10, where 0 means none of the time (poor) and 10 means all of the time (excellent). The secondary outcome was to compare the time to administration of the first analgesic post procedure, total postoperative opioid consumption (mg) at 24 hours, and duration of the procedure. Intraoperative hemodynamic stability and block-related and post-procedure complications were also assessed.

Statistical analysis

Data analysis was done using SPSS for Windows version 20.0 (IBM Corp., Armonk, NY). The data collected during the study were analyzed using descriptive statistics, such as percentage, mean, median, range, and standard deviation. Chi-square was used to find the association between attributes. Normality of the data was checked by the Kolmogorov-Smirnov test. For non-normality distributed data, the Mann-Whitney U test was used to compare the two groups. All statistical tests were conducted at a two-tailed level of significance (p ≤ 0.01 and p ≤ 0.05).

## Results

A total of 36 patients were enrolled in the study and were assigned alternately to one of the two groups after satisfying the inclusion and exclusion criteria, i.e., the TSA-ESPB group and the conscious sedation group. Table 1 demonstrates the baseline demographic data of the study subjects, which were comparable between the two groups, which had 18 patients each. The most common diagnosis was tuberculosis, followed by malignancy in both groups.

**Table 1:** Baseline characteristics of study subjects. ASA: American Society of Anesthesiologists; ESPB: erector spinae plane block.

Variables	ESPB group (n = 18)	Conscious sedation group (n = 18)	p-value	Chi-square test
Age (years)	52.33 ± 14.09	51.28 ± 17.24	0.81	1.54
Gender (female/male)	5/13	3/15	0.42	0.64
BMI (kg/m^2^)	23.11 ± 2.08	22.17 ± 2.18	0.37	0.8
Comorbidities, n (%)				
Hypertension	7 (38.9)	7 (38.9)	1	0.0
Diabetes mellitus	2 (11.1)	3 (16.7)	0.63	0.23
Chronic kidney disease	1 (5.6)	1 (5.6)	1	0.0
Epilepsy	1 (5.6)	0	0.31	1.02
ASA physical status (1/2/3)	2/12/4	2/13/4	0.26	0.62
Final diagnosis, n (%)				
Tuberculosis	13 (72.2)	11 (61.1)	0.14	6.83
Malignancy	2 (11.1)	4 (22.2)		
Empyema	2 (11.1)	0		
Non-specific	0	3 (16.7)		
Inconclusive	1 (5.6)	0		

**Figure 1:**
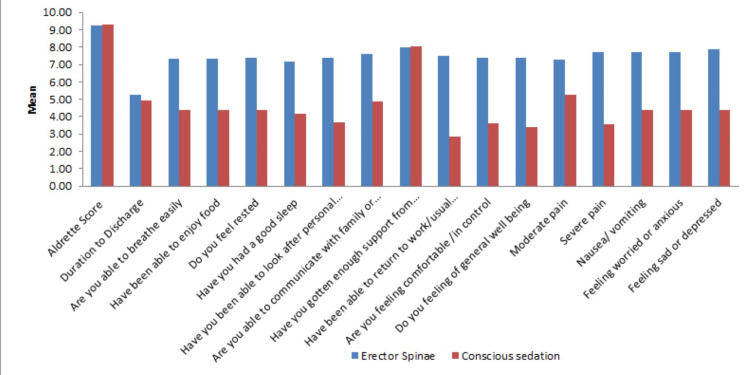
Graph comparing primary outcomes between the ESPB group and the conscious sedation group. ESPB: erector spinae plane block.

The primary and secondary outcomes of the study subjects are shown in Table 2 and Figure 1. The Aldrete score was comparable in both groups. Time to first analgesic was 11.69 ± 6.93 hours and 2.39 ± 1.69 hours, total opioid consumption at 24 hours was 50 ± 10 mg vs. 75 ± 15 mg, and total QoR-15 score was 112.83 ± 27.16 and 65.78 ± 22.13 in the TSA-ESPB and conscious sedation groups, respectively, which was statistically significant (p = 0.0001). Every component of part A and part B of the QoR-15 questionnaire showed statistical significance, including moderate to severe pain, between the two groups, except for getting support from hospital doctors and nurses, which was the same for both groups. The mean duration of the procedure in the TSA-ESPB group was much less than that of the conscious sedation group (64.72 vs. 93.89 minutes, p = 0.0001), which was statistically significant.

**Table 2:** Primary and secondary outcomes of study subjects. ESPB: erector spinae plane block; QoR-15: Quality of Recovery-15.

Variables	ESPB group (n = 18)	Conscious sedation group (n = 18)	p-value	Mann-Whitney U (z)
Primary outcome				
QoR-15 score	112.83 ± 27.16	65.78 ± 22.13	0.0001	4.25
Secondary outcome				
Time to first analgesic (hours)	11.69 ± 6.93	2.39 ± 1.69	0.0001	4.48
Total postoperative opioid consumption (mg) at 24 hours	50 ± 10	75 ± 15	0.001	4.21
Duration of procedure (minutes)	64.72 ± 12.06	93.89 ± 24.29	0.0001	4.56
Aldrete score	9.22 ± 0.43	9.28 ± 0.46	0.7	0.38

There were no statistically significant differences in the intraoperative lowest saturation, highest and lowest heart rate, systolic and diastolic blood pressure (BP), hypotension requiring intervention, and thoracoscopy complications in both groups, as shown in Table 3. No block/anesthesia-related complications were reported in either group.

**Table 3:** Intraoperative monitoring and adverse events. ESPB: erector spinae plane block; BP: blood pressure; ICD: intercostal drainage.

Variables	ESPB group (n = 18)	Conscious sedation group (n = 18)	p-value	t-value
Lowest saturation (%)	96.78 ± 2.56	96.67 ± 1.24	0.86	0.16
Highest heart rate (bpm)	113.89 ± 14.2	108.33 ± 9.24	0.17	1.39
Lowest heart rate (bpm)	89.72 ± 10.07	93.33 ± 10.29	0.29	1.06
Highest systolic BP (mmHg)	121.11 ± 9	118.89 ± 8.32	0.44	0.76
Lowest systolic BP (mmHg)	102.22 ± 10.03	107.78 ± 7.32	0.06	1.89
Highest diastolic BP (mmHg)	76.67 ± 5.94	76.11 ± 5.02	0.76	0.3
Lowest diastolic BP (mmHg)	71.67 ± 9.85	71.67 ± 7.86	1	0.0
Block complications	0	0		Chi-square test
Hypotension requiring intervention, n (%)	2 (11.1)	0	0.14	2.11
Thoracoscopy complications, n (%)				
Re-expansion pulmonary edema	0	1 (5.6)	0.47	3.54
Subcutaneous emphysema	5 (27.8)	3 (16.7)		
Minimal hemorrhage	0	1 (5.6)		
Prolonged ICD	1 (5.6)	0		

## Discussion

Following an extensive review of the available literature, to the best of our knowledge, this study is the first to compare TSA followed by ESPB with conscious sedation for MT using a patient-centered outcome measure, QoR-15, as the primary outcome. We demonstrated a clinically significant delay in the administration of the first analgesic, better QoR-15 scores at 24 hours, shorter duration of the procedure, and lower morbidity in terms of side effects like pain, nausea, and vomiting among patients in the ESPB group without any anesthesia-related complications.

A variety of measurement tools have been developed to measure the quality of recovery from the patient’s perspective. We used the QoR-15 score as it is an internationally validated tool that provides a valid, extensive, and efficient evaluation of a patient’s postoperative recovery [9]. In our study, for the conscious sedation group, we used lidocaine 1% as a local anesthetic [11] and a combination of a narcotic (fentanyl) and a benzodiazepine (midazolam) to achieve further analgesia, as used by Shojaee et al. [4].

Studies have shown that MT is associated with significant procedural and postoperative pain of varying intensity and duration [1,12]. Post-procedure pain stemming from pleural irritation and damage to the intercostal nerves and muscles is linked to substantial morbidity. This was evident in our study, where the duration of the procedure was significantly longer in the conscious sedation group. Effective analgesia during the procedure made the patients more cooperative, resulting in a shorter procedure duration in the TSA-ESPB group. Additionally, the time to first analgesia post procedure was much shorter, and the QoR-15 score was significantly lower in the conscious sedation group, indicating notable postoperative pain in this group. The surgical trauma and resultant pain stimulus of MT, particularly with the rigid thoracoscope, are highly comparable to surgical pain in VATS [4,11-13], highlighting the greater need for sedation/analgesia [8,14]. For MT, various strategies for pain management have been documented in the literature, combining a benzodiazepine with opioids or using titrated propofol infusions with or without opioids, and intrapleural lignocaine installation [4,12,15,16], emphasizing that one size does not fit all when it comes to the combination of anesthetic drugs in MT [17].

Studies have shown that complications occurring during endoscopy, such as hypoxemia, hypoventilation, apnea, and hypotension, and the need for airway rescue are not related to the procedure itself but rather to sedation [18,19]. To overcome the above complications, various regional anesthetic techniques have been used in thoracic procedures in the recent past to provide more effective thoracic analgesia in the peri and postoperative period like ESPB [6], serratus anterior plane (SAP) block [8], paravertebral block [20], midpoint transverse process to pleura blocks [21], and multi-level intercostal nerve blocks [22] with promising results.

**Figure 2:**
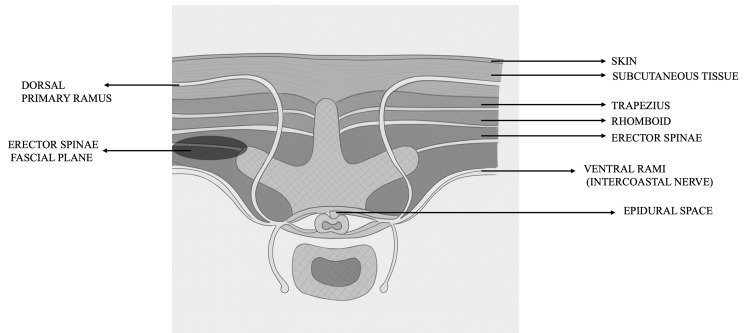
Schematic illustration of the optimal plane of the ESPB ("deep" to the erector spinae muscle). ESPB: erector spinae plane block. Figure credits: Sudeeksha P and Archana Baburao.

**Figure 3:**
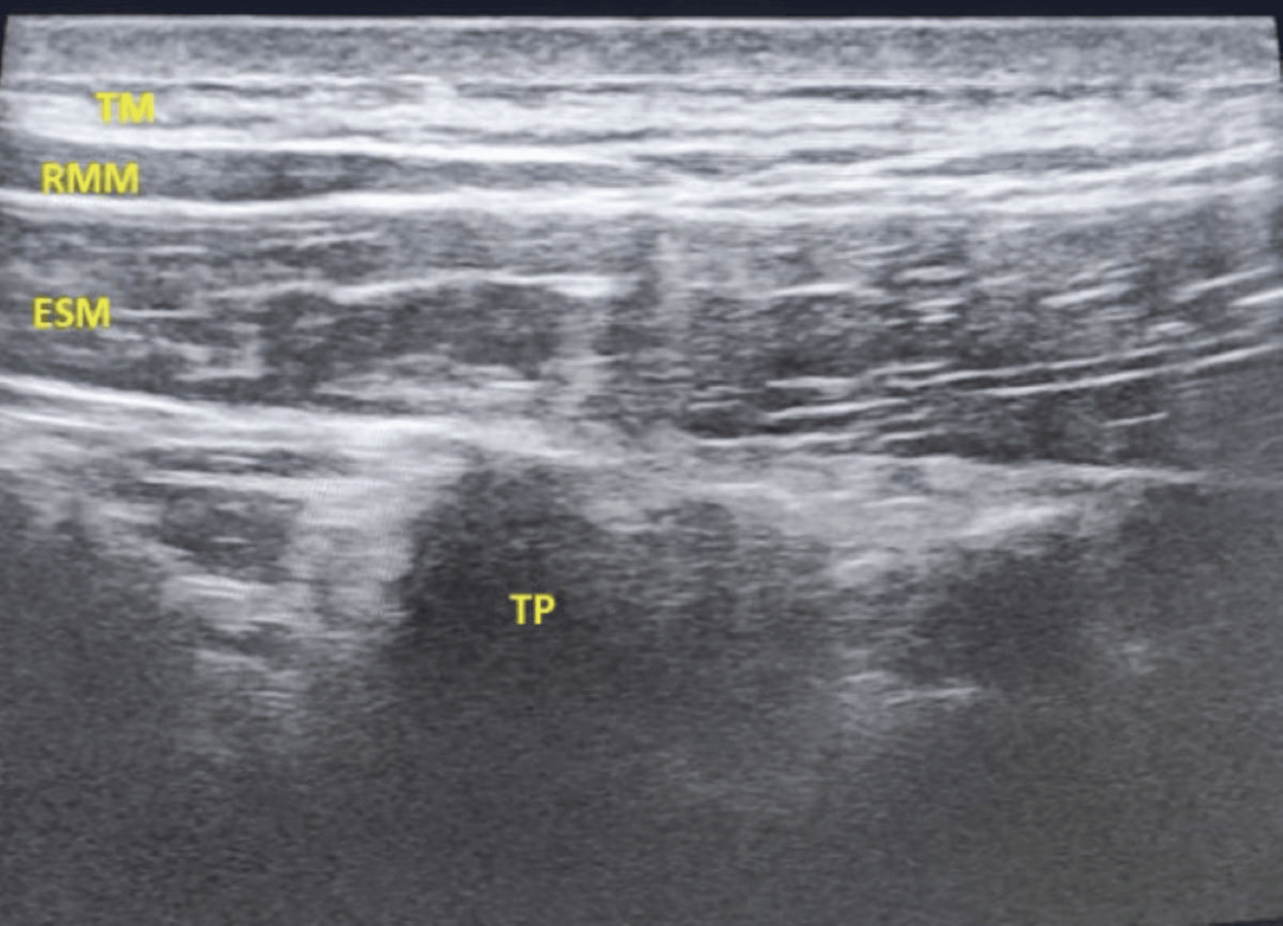
Sonoanatomy of the erector spinae muscle. TP: transverse process; TM: trapezius muscle; RMM: rhomboid major muscle; ESM: erector spinae muscle.

First described by Forero et al. in 2016 [6], ESPB is an interfascial plane block that involves the deposition of local anesthetic (LA) in the fascial plane between the erector spinae muscle and the tip of the transverse processes of the vertebrae. The optimal plane for injection in our study was "deep" to the erector spinae muscle (Figure 2) as this will deposit LA closer to the dorsal and ventral rami, thus permitting extensive cranio-caudal spread and coverage of multiple dermatomes, resulting in both somatic and visceral analgesia. Easily recognizable sonoanatomy (Figure 3), including the transverse process and no structures at risk of needle injury in the immediate vicinity, makes the ESPB a safe and simple procedure [19]. Single-site ESPBs are usually given at the spinal level ranging between T5 and T7; in our study, the block was given at the level of T6. Studies have compared ESPB with SAP block for thoracic surgery; ESPB demonstrated superior analgesic efficacy, lower morbidity, and superior quality of recovery at 24 hours in the postoperative period [6]. The PROSPECT guidelines also recommend regional analgesic techniques such as paravertebral block and ESPB for pain management in patients undergoing VATS [23].

The duration of analgesia after a single-shot ESPB lasts for 10-16 hours and does not provide long-term analgesia in the postoperative period [24]. This was evident in the study by McPherson et al. [25], wherein pain scores (1 - least amount of pain and 10 - the worst pain) significantly increased over time from 0.66 during the periprocedural period to 1.56 in the recovery room, 3.56 at three to 12 hours post discharge, and 5.56 on the first postoperative day, requiring the use of opioids. The quality of recovery is impaired by the early postoperative pain, which is poorly controlled, thus increasing the risk of postoperative pulmonary complications.

To provide prolonged analgesia in the postoperative period, continuous infusion techniques using ultrasound-guided insertion of catheters into the ESP have been employed. However, they pose complications such as bleeding, infection, cumbersome insertion, and difficulty in management and maintenance [26]. Thoracic spinal anesthesia has served as the sole anesthetic technique in thoracic, cardiac, and abdominal surgeries for patients with severe respiratory disease due to several advantages: an awake, spontaneously breathing patient, stable hemodynamic parameters in the perioperative period, reduced morbidity, improved intra- and postoperative pain control, and subsequent early hospital discharge [27,28]. Therefore, we combined TSA with ESPB to prolong analgesia duration and reduce morbidity in the extended postoperative period. This was supported by the results of a recent multicentric study by Deshpande and colleagues, which demonstrated excellent efficacy of TSA along with unilateral ESPB in patients who underwent unilateral modified radical mastectomy and axillary dissection with an acceptable safety profile [29]. In our study, TSA was administered at the level of T6/T7, since the distance between the dura mater and spinal cord is greatest in the midthoracic region, which can be further increased in the head-down posture, resulting in sensory block distribution between T1 and T11 [30]. Our study is the first of its kind, wherein we compared combined TSA and unilateral ESPB with conscious sedation for patients undergoing MT, and we could demonstrate that the former technique was safe, efficacious, and well tolerated by patients with overall lesser morbidity. However, one should be aware of the recognized risks of TSA, like high spinal block, hypotension, and neurological injury.

Our study has several limitations. It is a single-center study with a small sample size (sample size was based on feasibility since this is a pilot study), a lack of randomization, and allocation bias, making it difficult to generalize the results. Additionally, we did not calculate the costs involved in the procedure. Despite these limitations, this technique has shown better patient outcomes; therefore, there is a need for larger randomized controlled trials.

## Conclusions

The present study suggests that the combination of TSA and ESPB represents a promising anesthetic approach for MT. This technique was associated with prolonged postoperative analgesia, improved quality of recovery at 24 hours, reduced procedure duration, and a lower incidence of perioperative morbidity. These findings indicate potential advantages in terms of patient comfort, functional recovery, and procedural efficiency, underscoring its potential safety benefits, while no complications were observed. However, larger, well-designed randomized controlled trials are required to confirm these results and to establish the reproducibility, long-term safety, and clinical applicability of this combined anesthetic strategy in routine thoracoscopic practice.
